# Metabolite Profiles of Sugarcane Culm Reveal the Relationship Among Metabolism and Axillary Bud Outgrowth in Genetically Related Sugarcane Commercial Cultivars

**DOI:** 10.3389/fpls.2018.00857

**Published:** 2018-06-25

**Authors:** Danilo A. Ferreira, Marina C. M. Martins, Adriana Cheavegatti-Gianotto, Monalisa S. Carneiro, Rodrigo R. Amadeu, Juliana A. Aricetti, Lucia D. Wolf, Hermann P. Hoffmann, Luis G. F. de Abreu, Camila Caldana

**Affiliations:** ^1^Brazilian Bioethanol Science and Technology Laboratory, Centro Nacional de Pesquisa em Energia e Materiais, Campinas, Brazil; ^2^Genetics and Molecular Biology Graduate Program, University of Campinas, Campinas, Brazil; ^3^Department of Biotechnology and Plant and Animal Production, Center for Agricultural Sciences, Federal University of São Carlos, São Carlos, Brazil; ^4^Department of Genetics, Luiz de Queiroz College of Agriculture, University of São Paulo, Piracicaba, Brazil; ^5^Max-Planck Partner Group, Brazilian Bioethanol Science and Technology Laboratory, Centro Nacional de Pesquisa em Energia e Materiais, Campinas, Brazil

**Keywords:** sugarcane, breeding, metabolome, bud outgrowth, metabolic network

## Abstract

Metabolic composition is known to exert influence on several important agronomic traits, and metabolomics, which represents the chemical composition in a cell, has long been recognized as a powerful tool for bridging phenotype–genotype interactions. In this work, sixteen truly representative sugarcane Brazilian varieties were selected to explore the metabolic networks in buds and culms, the tissues involved in the vegetative propagation of this species. Due to the fact that bud sprouting is a key trait determining crop establishment in the field, the sprouting potential among the genotypes was evaluated. The use of partial least square discriminant analysis indicated only mild differences on bud outgrowth potential under controlled environmental conditions. However, primary metabolite profiling provided information on the variability of metabolic features even under a narrow genetic background, typical for modern sugarcane cultivars. Metabolite–metabolite correlations within and between tissues revealed more complex patterns for culms in relation to buds, and enabled the recognition of key metabolites (e.g., sucrose, putrescine, glutamate, serine, and myo-inositol) affecting sprouting ability. Finally, those results were associated with the genetic background of each cultivar, showing that metabolites can be potentially used as indicators for the genetic background.

## Introduction

Plants have an extraordinarily complex metabolism, and a comprehensive understanding on how it operates pose a challenge due to the coordination among various biochemical processes in specialized tissues and subcellular compartments (Lunn, 2007; Sweetlove and Fernie, 2013). Their sessile nature adds an extra layer of difficult, as there is a constant need to adjust to changes in the surrounding environment (Jaillais and Chory, 2010; Kooke and Keurentjes, 2012). This significant level of organization allows the production of a plethora of chemical compounds, which differ in their properties (e.g., size, polarity, stability, and quantity) and biological functions, representing a readout of the physiological status of a cell. Traditionally, plant metabolomics studies have focused on elucidating the function and regulation of particular biosynthetic routes involving a number of metabolites (Stitt et al., 2010; Fernie and Tohge, 2017). However, advances in large-scale automated analytical platforms have increasingly enabled high-throughput detection of metabolites, allowing the elucidation of metabolic networks in terms of structure and connectivity and/or bridging the genotype-to-phenotype gap to elucidate certain biological processes. Although knowledge about the role of specific enzymes was extended by targeted reverse genetics approaches (Alex et al., 2004; Tohge et al., 2005; Zheng et al., 2005; Seki et al., 2011; Goulet et al., 2015), duplication of enzymes and their different subcellular localization hampers metabolic engineering modifications relying on a single transgenic (Huang et al., 2010; Qin et al., 2011; Ren et al., 2014; Fernie and Tohge, 2017). Means to surpass this problem include the use of natural/genetic variance to enhance our understanding about the genetic architecture of metabolic traits and monitor gene-phenotype combinations in a wide range of plant species (e.g., Arabidopsis, tomato, and rice) or important agronomic traits such as fruit composition (Bernillon et al., 2013; Monti et al., 2016), grain yield (Obata et al., 2015; Dan et al., 2016), and tolerance to abiotic stresses (Glaubitz et al., 2014; Todaka et al., 2017; Sprenger et al., 2018).

As metabolism is strongly influenced by interactions between the environment and genetic regulation, there is a limitation to extrapolate the complete picture of plant metabolomes by evaluating a single condition (i.e., developmental stage, genetic background and environment) (Soltis and Kliebenstein, 2015). Furthermore, apart from having their biosynthesis and accumulation in a tissue-specific manner, metabolites can be produced and transported across tissues and/or organs to mediate certain biological processes. One example of this kind of regulation is the fate of axillary buds, which is governed by a complex interplay among environmental factors, genetic background and endogenous metabolites (Huang et al., 2012). Metabolite signals arising from other parts of the plant such as shoots or stems are sensed prior to trigger systemic responses that will promote bud outgrowth (Dun et al., 2012; Barbier F.F. et al., 2015; Brewer et al., 2015). Several hormones have been long recognized as the main signaling molecules in this process (Umehara et al., 2008; Domagalska and Leyser, 2011; Durbak et al., 2012). However, the availability of sugars, especially sucrose, was recently found to be crucial for bud outgrowth release prior to alterations in hormone levels (Mason et al., 2014; Barbier F. et al., 2015). Manipulation of sucrose supply via decapitation or defoliation was able to promote or suppress bud outgrowth, respectively (Mason et al., 2014; Barbier F. et al., 2015; Fichtner et al., 2017). Interestingly, dormant buds present a transcriptional response related to carbon starvation that seems to be conserved among different species (Tarancón et al., 2017). Primary metabolites, such as sugars and amino acids, are integral parts of sophisticated signaling networks linking the energetic status and external cues to regulate growth accordingly (Nunes-Nesi et al., 2010; Smeekens et al., 2010; Xiong et al., 2013; Chellamuthu et al., 2014; Yadav et al., 2014). Collectively, this new information placed primary metabolites as essential molecules with more immediate roles in bud development and outgrowth.

The regulation of axillary bud outgrowth is crucial for crops in which either vegetative propagation or tillering are important traits, as it is the case of the perennial C4 grass sugarcane (*Saccharum* × *officinarum*). In sugarcane, axillary buds are also naturally in a dormant state (Jain et al., 2009), however, when segments of the culms containing portions of internode and node with embryo roots and at least one viable bud are isolated from the plant body and placed into soil, bud outgrowth is released and a new plant is generated. Sugarcane is capable of accumulating impressive amounts of sucrose in its stems, in a very complex and dynamic process characterized by a continuous cycle of synthesis and degradation (Whittaker and Botha, 1997; Zhu et al., 1997; Botha and Black, 2000; Rose and Botha, 2000), which involves various enzymes and their isoforms. There is a gradient of sucrose accumulation along the stem, with younger internodes containing less sucrose than older internodes (Zhu et al., 1997; Pereira et al., 2017). Interestingly, the stored carbon in the form of sucrose is used for bud outgrowth and formation of a new sugarcane plant (O’Neill et al., 2012). Due to the fact that sucrose was shown to be crucial for bud outgrowth in other species (Mason et al., 2014; Barbier F. et al., 2015; Kebrom and Mullet, 2015), it remains to be elucidated whether the remarkably high levels of sucrose or other components of the primary metabolism are important to promote bud outgrowth release in sugarcane. The complex genetic architecture of sugarcane (e.g., high polyploidy, high heterozygosity, large amount of repetitive sequences, aneuploidy, and large genome size) (Zhang et al., 2012) has hampered the use of genetic information to dissect biological mechanisms in this species (de Setta et al., 2014; Song et al., 2016; Riaño-Pachón and Mattiello, 2017; Hoang et al., 2017). All these characteristics make the application of metabolomics a great alternative for investigating complex agronomic traits such as sprouting potential.

In the present study, we assessed the metabolic profile of two tissues involved in the sprouting potential, namely culm and bud, from 16 highly planted sugarcane varieties from a Brazilian sugarcane breeding program (varietal census 2016/2017^1^). The cultivars studied herein rank among the most cultivated genotypes in the world as they cover about 65% of the sugarcane planted area in Brazil, the major sugarcane producer worldwide. These cultivars are therefore a worthy sample of sugarcane commercial genotypes with greater field performance. Our results demonstrate that the culm metabolism plays an important role as primary energy source to provide carbon skeletons and building blocks for protein synthesis for triggering bud outgrowth. Overall, our results suggest that both factors, genetic background and bud sprouting rates, jointly influenced the metabolite profile of sugarcane, opening perspectives for the use of metabolomics to assist sugarcane breeding programs.

## Materials and Methods

### Plant Material

A collection of 16 relevant genotypes was chosen from the leading Brazilian sugarcane-breeding program The Inter-University Network for the Development of Sugarcane Industry (RIDESA) (**Supplementary Table S1**). Out of 16, the varieties RB867515, RB966928 and RB92579 cover 42% of total sugarcane fields in the country (2016/2017 varieties census: see footnote text 1). Sugarcane breeding programs have been indirect selected genotypes with high sprouting potential by choosing experimental plots with higher density and stalk yield. Consequently, the current commercial cultivars have low variability to this trait and tend to present medium to high sprouting potential under field conditions (Cargnin et al., 2008). The sugarcane breeding programs rely generally on a limited number of elite plant material as parental lines. Therefore, these 16 selected genotypes were also used as a proof of concept to evaluate whether even under a narrow genetic basis metabolic profiles could be used to discriminate their metabolic status. Information on the parents of the selected genotypes was recorded from RIDESA database, the pedigree tree (**Supplementary Figure S1**) was drawn using the R packages synbreed (Wimmer et al., 2012) and diagram (Soetaert, 2017). The degree of kinship among the cultivars is represented as the coefficient of relationship between the individuals (Wright, 1922) computed using AGHmatrix package (Amadeu et al., 2016; **Supplementary Figure S2**).

### Experimental Conditions

Field and greenhouse experiments were conducted at the Federal University of São Carlos (UFSCar)/RIDESA in Araras, São Paulo, Brazil located at 22°21′25′′ S, 47°23′03′′ W, about 611 m above sea level; in a typic eutroferric red latosol soil. Mature sugarcane plants (approximately 11 months old) were decapitated 24 h before the harvest in the field, to facilitate the loss of apical dominance. After that, three stems of each genotype from independent plants were randomly harvested around 2 h after dawn. For each stem, internodes were counted and divided into three parts. Due to variations in sprouting performance throughout the stem according to bud position, only internodes belonging to the middle portion of the stem were further cut close to the bud, and had their diameter and weight measured to guarantee uniformity. Considering the existence of a sucrose gradient as well as different developmental stages along the sugarcane stem, the selection of the middle third portion of this organ would allow a better comparison among the genotypes. It is worth mentioning that usually entire sugarcane stems are planted in commercial field environments.

This material, also known as setts, was used for both metabolite profiling analysis and sprouting performance evaluation. In case of metabolite profiling, buds and the region of the culm in which they were inserted in were precisely isolated with the help of a scalpel and cork borer, respectively. A total of 3 biological replicates (representing three independent stalks), each containing a pool of three individual buds or culms, was collected. After the harvesting process, which took approximately 5 min per genotype, tissues were immediately frozen into liquid nitrogen and stored at -80°C for metabolic profiling analysis.

Setts were planted with buds oriented toward the light into 200 ml pots containing commercial substrate Plantmax^®^ for sprouting evaluation. Since sugarcane initial development is sensitive to soil water content and changes in temperature (Oliveira et al., 2001; Singels and Smit, 2002; Smit, 2011), the experiment was performed in the greenhouse during May 2016 with automated irrigation system (six times along the diel cycle) and the temperature was set to 35 and 29°C before and after sprouting, respectively. Each genotype was planted in three completely randomized trays containing 24 individuals each.

### Sprouting Rate Evaluation

Sprouting performance was assessed in the greenhouse by monitoring bud outgrowth during the first 14 days after planting. Even considering the lack of synchronization in bud outgrowth release and their potential to be viable over longer terms, dormant buds would hardly become seedlings under field conditions after this period. Sprouting was considered successful when the seedling stem crossed the soil surface (a layer of 2 cm of soil over the bud) and was able to issue the first leaf. In order to classify the genotypes according to the sprouting rate, a descriptive quartile analysis was performed, in which varieties belonging to the top or down 25% of data distribution were considered with high or low sprouting potential, respectively. To further improve the classification of the genotypes belonging to the middle 50% quartile of data distribution, a second quartile analysis was performed to distinguish them as intermediate-low or -high sprouting potential. For assessing the variances, a parametric test (*F*-test) was applied to all genotypes at 5% of significance levels.

### Metabolite Profiling Analysis

Prior to metabolite profiling analyses, sugarcane tissues were ground to a fine powder in liquid nitrogen and aliquots of 20 or 50 mg for culms and buds, respectively, were used for metabolite extraction, following the methodology described by Giavalisco et al. (2011). A fraction of 100 μl from the organic phase was dried and derivatized as described in Roessner et al., 2001. Afterwards, 1 μl of the derivatized samples was analyzed on a Combi-PAL autosampler (Agilent Technologies GmbH, Waldbronn, Germany) coupled to an Agilent 7890 gas chromatograph and a Leco Pegasus 2 time-of-flight mass spectrometer (LECO, St. Joseph, MI, United States) in both split (1:40 and 1:65 for buds and culm, respectively) and splitless modes (Weckwerth et al., 2004). Chromatograms were exported from Leco ChromaTOF software (version 3.25) to R software. Peak detection, retention time alignment, and library matching using the Golm Database^2^ were performed using TargetSearch R package (Cuadros-Inostroza et al., 2009). Metabolites were quantified by the peak intensity of a selective mass. Metabolites intensities were normalized by dividing the fresh weight of each biological replicate, followed by the sum of total ion count and log2 transformation.

### Statistical Analyses

Statistical analyses and graphical representations were performed using R version 3.2.3^3^. Multivariate analyses, including PCA and PLS-DA, were carried out using mixOmics (Rohart et al., 2017) and pcaMethods (Stacklies et al., 2007) R packages. Correlation analysis, heatmap and network visualization were done using corrplot (Wei and Simko, 2017), d3heatmap (Cheng et al., 2018), and qgraph (Epskamp et al., 2012) packages, respectively.

## Results

### Commercial Sugarcane Varieties Displayed Mild to Low Variability in Bud Outgrowth Under Controlled Environmental Conditions

During the initial period of seedling establishment (14 days), an overall high sprouting homogeneity was observed among genotypes. The first quartile analysis allowed the classification of 31, 56, and 13% of the genotypes into low, intermediate, and high sprouting potential, respectively. To refine this classification, a second quartile analysis was performed only using the intermediate genotypes. However, due to the fairly homogenous sprouting ability of the genotypes observed in the present study, the subdivision of this group resulted only in 19 and 38% of genotypes with intermediate–low and intermediate–high sprouting potential, respectively. Out of the 16 selected genotypes, RB975375 and RB935744 were classified as high sprouting rate, whereas RB937570, RB975201, RB835486, RB966928, and RB72454 were considered with low sprouting potential (**Supplementary Figure S3**). Despite these major groups, the sprouting rate was in the range of 89–100% and no statistical differences among the genotypes were observed at level of 5% of significance. Due to the nature of vegetative propagation of this crop, breeding programs have indirectly favored genotypes with high sprouting rates. However, there is still a severe lack of synchronization of the bud outgrowth in the Brazilian field conditions, especially in areas susceptible to drought, leading to massive reduction in sprouting and consequently jeopardizing yield (Cargnin et al., 2008). Furthermore, the sprouting rate estimation was based on the establishment of a new plant, and although this is the measured trait in the field, which indirectly reflects the bud outgrowth performance, it does not enable the assessment of the internal factors involved in the control of bud release.

### Metabolite Profiling Revealed Differential Metabolic Responses of Genotypes in Distinct Tissues

**FIGURE 1:**
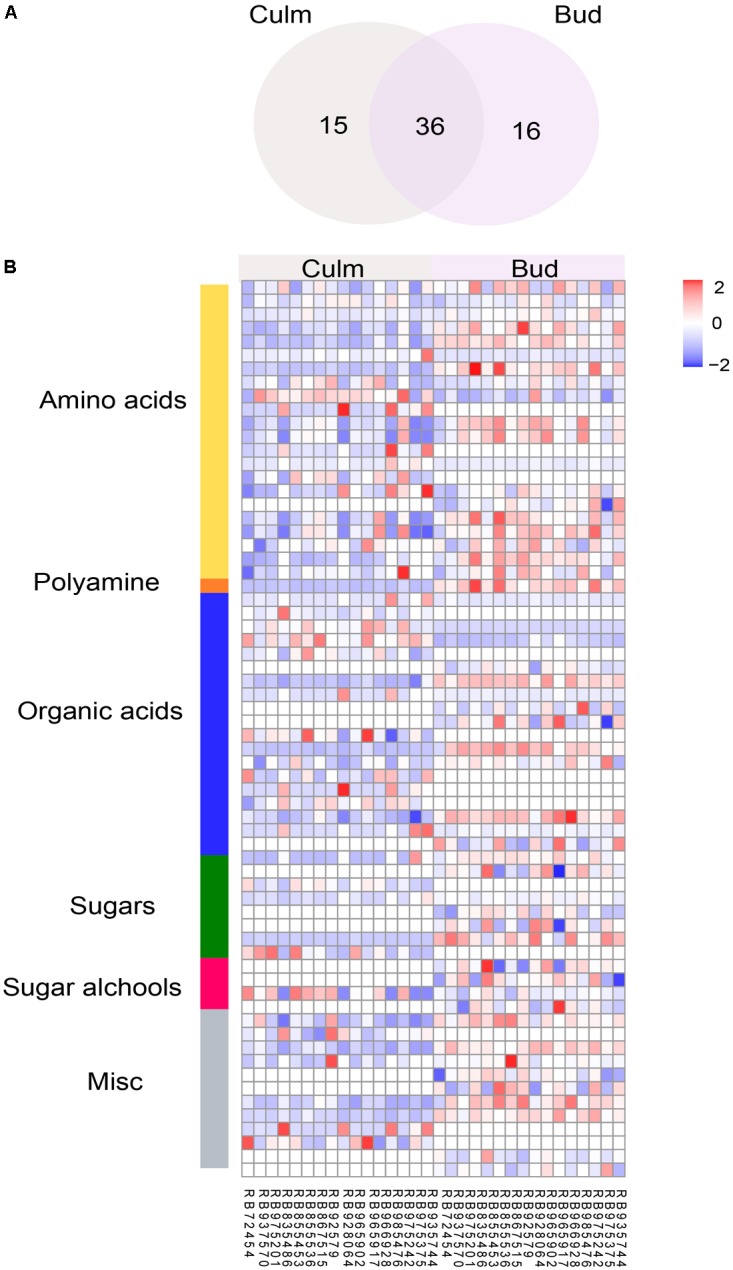
Metabolic composition of buds and culms in different sugarcane commercial cultivars. **(A)** Number of metabolites identified in culm, bud, and common to both tissues; **(B)** Differential abundance of metabolites in culms and buds according to their compound class. Each column represents the genotype mean, and each row represents a metabolite. Variations in the relative abundance of metabolites are displayed in blue (low) to red (high).

As it is challenging to morphologically and molecularly monitor the factors triggering bud outgrowth release in this crop, we next investigated whether the metabolite profiling could be a great tool to understand the control of bud outgrowth using GC-MS. This platform allows the assessment of molecules involved mainly in central metabolism, which was already reported to be closely linked to plant growth (Meyer et al., 2007; Lisec et al., 2008). Due to the fact that no significant differences were found in water content of the studied genotypes (data not shown), we used fresh weight for normalizing the metabolite levels. A major portion of the metabolites (76.7 %) was found in all samples. Due to the saturation of sucrose levels, the same samples were also injected in a split mode (diluted 1:40 or 1:65 for buds and culms, respectively) to accurately quantify this sugar. We detected a total of 66 metabolites with known structures (e.g., amino acids, sugar, sugar alcohols, organic acids, and polyamines), of which 16 and 15 were specifically identified in bud and culm, respectively (**Figure 1A**). **Figure 1B** shows a heatmap including the metabolite abundance of genotypes in each tissue and **Supplementary Table S2** summarizes the effect of individual metabolites. By applying ANOVA, we found that most metabolites were significantly affected by the genotype in both tissues (**Supplementary Figure S4**), indicating enough variability in metabolic features among genotypes even under a narrow genetic basis (**Supplementary Figures S1**, **S2**).

### Metabolite–Metabolite Correlations Provide New Insights on the Regulation of Metabolic Networks Intra- and Inter-Tissues

**FIGURE 2:**
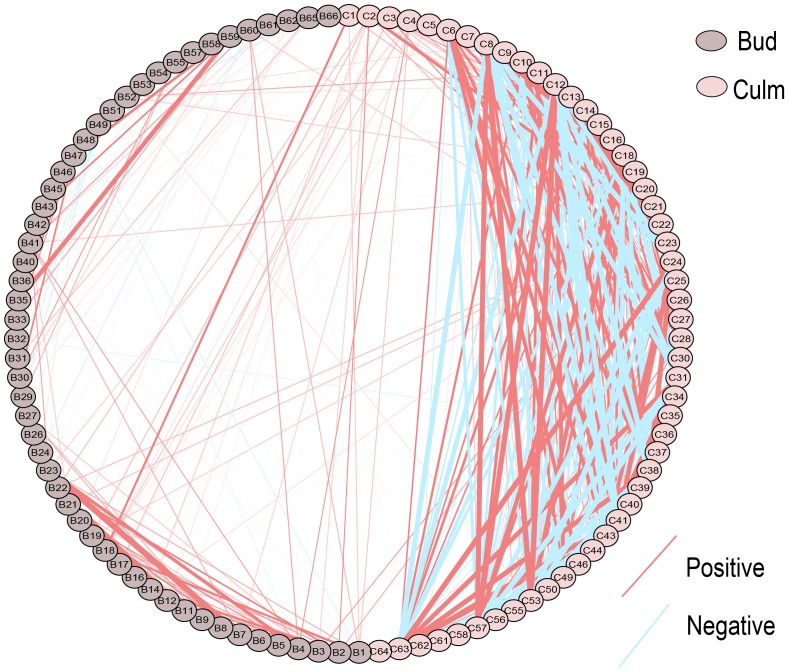
Metabolite–metabolite correlations within and between tissues in different sugarcane commercial cultivars. Metabolites are indicated by codes available at the **Supplementary Table S2**. Positive and negative correlations are represented in red and blue, respectively. Different color of nodes denotes distinct tissues.

To decipher the relationships among metabolites, we performed correlation-based network analysis using significant pairwise correlations (*r* ≥ 0.5, *p* ≤ 0.05) (**Figure 2**), which are summarized in **Supplementary Table S3**. As expected, metabolites belonging to the same biochemical pathway tended to present a high degree of connectivity as it was the case for valine, isoleucine and leucine (*r* > 0.8) in both tissues. In total, there were 148, 414, and 47 significant correlations among metabolites detected in buds, culms and between these two tissues, respectively, suggesting that the metabolite–metabolite correlations were diverse in the different tissues. An exception was a subnetwork containing positive correlations among the branched amino acids leucine, isoleucine and valine, and threonine, conserved in both tissues. Interestingly, those amino acids were only linked to each other and methionine in buds, whereas in culm this network became more complex. Apart from the branched amino acids and threonine, further highly positive connections were built among glutamine, serine and the sugars sucrose, fructose and myo-inositol in culms. This expanded subnetwork was negatively linked to another subnetwork including GABA, putrescine, benzoate, galacturonate, nicotinate, and a metabolite with similarity to itaconate, which in turn were all positively correlated to each other. The strongest negative correlation (*r* = -0.9679, *p* = 0.05) was between sucrose and putrescine that is one of the links between these two subnetworks in culms. Remarkably, the role of those two metabolites in controlling bud dormancy have been recently shown (Cui et al., 2010; Mason et al., 2014; Barbier F. et al., 2015). Interestingly, the compound similar to itaconate, which displayed the strongest positive correlation in culm with galacturonate (*r* = 0.977, *p* = 0.05) presented distinct correlations with amino acids in culms and buds. One example is the connection with glutamate that seems to be the opposite in both tissues. Glutamate and serine are the only metabolites that significant connect the bud and culm network. With respect to the bud, its network is much less interconnected when compared to culm. In addition to the amino acids subnetwork, another highly interconnected subnetwork containing galactose, quinate and sorbose (*r* > 0.83, *p* = 0.05) was identified. Altogether, these results revealed that the metabolic network of culm is more coordinately regulated than that of bud. A logical explanation is that culms constitute more active tissues with pivotal role as strong sinks, not only related to sucrose, but also including amino acids, which will act as primary energy source to provide carbon skeletons and building blocks for protein synthesis during bud outgrowth.

### Culm Metabolic Composition Is Important for Bud Outgrowth Performance

**FIGURE 3:**
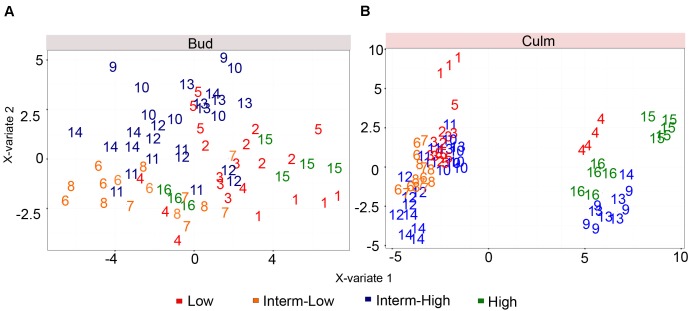
Partial least squares discriminant analysis (PLS-DA) score plots corresponding to a model using genotypes and metabolites as two latent variables. **(A,B)** represent the discriminant analysis of bud and culm, respectively. The different genotypes are referred as numbers from 1 to 16, listed in **Supplementary Table S1**. The color of the numbers denotes its sprouting group.

We also investigated whether the metabolic composition of both tissues among the selected commercial cultivars would have an impact on sprouting even under low trait variability. Based on the role of polyamines and sugars controlling axillary meristem dormancy and tillering/branching (Zheng et al., 2005; Ge et al., 2006; Purohit et al., 2007; Cui et al., 2010; Mason et al., 2014; Barbier F. et al., 2015), we hypothesized that the metabolic composition of the culm could be one of the key factors determining bud outgrowth. Although our analysis was restricted to few compounds of the primary metabolism, it covers key metabolites known to play fundamental roles in promoting sink to source transitions and plant growth. A partial least squares discriminant analysis (PLS-DA) was applied in an attempt to understand the role of culms in bud outgrowth (**Figure 3**). Our results showed that the bud metabolomic data solely did not result in a good separation among genotypes according to the sprouting potential (**Figure 3A**). One plausible reason is that different genotypes might be at distinct stages of dormancy, which could exert influence on their metabolism hampering the discrimination based on the metabolome. Furthermore, as the metabolic activities of mature culms are committed to sucrose storage and reduced in comparison to fast growing stages of the plant life cycle, we cannot exclude the possibility that changes in metabolic contents among cultivars are partially masked at this specific phase. In contrast to bud, the PLS-DA revealed a significant difference in the culm metabolism for low and high sprouting rate at least for the most contrasting genotypes (**Figure 3B**).

**FIGURE 4:**
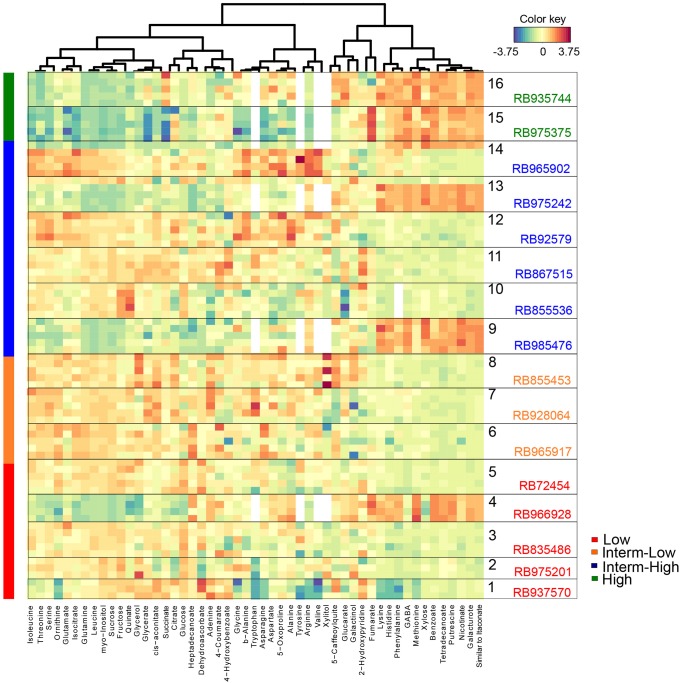
Heat map of metabolites in culms selected by the PLS-DA VIP score. Each row represents a metabolite identified in at least three biological replicates (the number of squares represent the exact number of replicates), whereas columns represent the metabolic abundance among genotypes. Changes in the abundance of metabolites from the overall mean concentration for each genotype are shown in blue (low correlation) or red (high correlation).

To identify the metabolites responsible for the separation among genotypes, a cluster image analysis was performed by building a similarity matrix with the PLS-DA results (**Figure 4**). A total of five well-defined groups was obtained and although there was no clear association in three of the clusters with respect to sprouting rate, two groups presented a very clear trend in relation to bud outgrowth performance. Interestingly, the levels of several metabolites including lysine, histidine, phenylalanine, GABA, methionine, xylose, benzoate, tetradecanoate, putrescine, nicotinate, galacturonate, the compound similar to itaconate, putrescine, nicotinate, benzoate, and galacturonate seemed to correlate positively with the high sprouting rate genotypes. In contrast, the levels of glycerol, quinate, fructose, sucrose, myo-inositol, leucine, glutamine, isocitrate, glutamate, ornithine, serine, threonine, and isoleucine appeared to have a negative impact on bud outgrowth. Taken together, our results showed that the culm, but not the bud metabolome, enabled the discrimination of the genotypes based on their sprouting performance only among the most contrasting cultivars. Furthermore, the culm metabolism turned up important to determine bud outgrowth efficiency as shown by the correlation of contrasting genotypes with antagonistic metabolites such as sugars and polyamines, this latter suggested as a signaling mediator in bud dormancy, and also by the presence of glutamate and serine, both involved in the connection of culm and bud networks.

### Sugarcane Metabolome Reflects Sprouting Rate and the Genetic Relatedness of Commercial Genotypes

**FIGURE 5:**
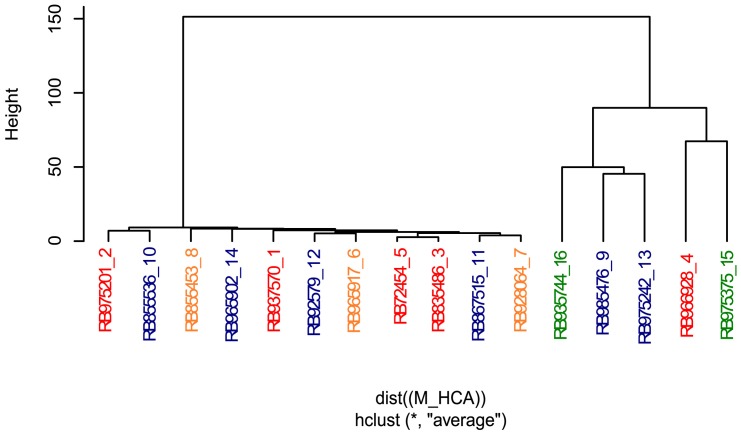
Hierarchical cluster analysis revealed that metabolite profiling from culms discriminates sugarcane commercial cultivars. Genotypes are color-coded as follows: red, orange, blue, and green represent low, intermediate-low, intermediate-high, and high sprouting groups, respectively.

As the culm metabolome permitted to rank at some extend the genotypes according to the sprouting rate, we next investigated how central metabolism was influenced by the genetic background of the selected cultivars in the conditions of this experiment. For that, we compared the hierarchical clustering analysis (HCA) considering only the culm metabolome (**Figure 5**) to numerator relationship matrix (**Supplementary Figure S2**) obtained from pedigree information shown in **Supplementary Figure S1**.

The HCA analysis revealed the presence of two defined clusters (**Figure 5**). The first cluster represents a very similar group of eleven genotypes with low to moderate-high sprouting rates. Interestingly, pairs of individuals with a relationship coefficient of 0.5 (parent-offspring or full-sib) in the numerator relationship matrix (**Supplementary Figure S2**) tended to be kept in this HCA cluster. This cluster was mainly composed of individuals genetically related to the genotype RB72454, which was used as parental of several crossings of this panel (**Supplementary Figure S1**). The mismatch individual, RB975375, displayed 100% of sprouting rate. The other exception was the genotype RB835486, which clustered together with RB72454 despite their complete absence of relatedness as per pedigree information (**Supplementary Figure S1**). Interesting to note that RB835486 displayed the third lowest sprouting rate (93.1%), which main explain its placement at this first cluster.

The second cluster represents cultivars with high and moderate-high sprouting rates (RB935744 and RB975375 – high; RB985476 and RB975242 – moderate-high) with the exception of cultivar RB966928 that presents low sprouting rate. All the cultivars located in this cluster tended to display parentage coefficient below 0.25, among themselves and with other cultivars of this panel (**Supplementary Figure S2**), according to the information from our pedigree. This fact is reflected in the height of the HCA dendrogram, which confirms their relative weaker genetic relationship.

It is worth to mention that our pedigree estimate is incomplete due the lack of information about the fathers of cultivars obtained from multiparental crosses. Besides, the estimation of the kinship matrix (**Supplementary Figure S2**) could also be improved using informative molecular markers, which were out of the scope of this work. Despite this, the numerator relationship matrix (**Supplementary Figure S2**) revealed that the parentage coefficient was already high among cultivars (average of 0.114), suggesting a close relationship among cultivars (i.e., such value is almost the coefficient between cousins, 0.125). Apart from RB92579 and RB975242, all the clones share some degree of relatedness with at least one individual in the panel (**Supplementary Figures S1**, **S2**). Overall, our results suggest that both factors, genetic background and bud sprouting rates, jointly influenced the metabolomic profile of sugarcane revealed by HCA when evaluated under a single environmental condition.

## Discussion

Metabolomics has been widely used as a powerful tool to elucidate mechanisms involved in metabolic regulation as well as bridging the gap between genotype and phenotype (Saito and Matsuda, 2010; Kusano et al., 2011; Kumar et al., 2017). Over the last decade, metabolomics has been also used in association with natural variation to unravel the genetic architecture of several agronomic traits controlled by metabolism (Meyer et al., 2007; Toubiana et al., 2012; Witt et al., 2012). Although these studies have provided many insights into biological properties, the complexity of the metabolome and its dependency on the environment, genetics and development, precludes the generation of a full complete picture (Soltis and Kliebenstein, 2015). In this study, we minimized this complexity by fixing the environmental conditions to assess whether the primary metabolism of two organs involved in the sprouting potential is regulated to trigger bud outgrowth in 16-highly planted sugarcane cultivars, representing a good sampling of commercial cultivars with overall good field performance, including good sprouting rate. Under our experimental conditions (controlled temperature and water availability), the selected sugarcane genotypes presented low variability in their sprouting rate (about 10%). Optimal sprouting conditions rarely occur in important production areas, when buds are often exposed to several abiotic constraints. One example is the Brazilian central region, in which the impact of limiting environmental conditions resulted in sprouting rates ranging from 29,17 to 76,92% among 8 commercial cultivars. This work evaluated 4 cultivars (RB867515, RB855453, RB835486, RB855536) also studied herein, but only RB8555453 and RB855536 presented sprouting ratios over 70% under those environmental conditions (Cargnin et al., 2008). Furthermore, it is important to mention that a mix of internodes from top, middle and bottom portions of the stem is planted in commercial fields. In our experimental setup, we selected only internodes belonging to the middle part of the stem to minimize the variation in sprouting rate dependent on developmental stage and bud position among the varieties (Manhães et al., 2015; Baracat-Neto et al., 2017).

It is widely known that there is a gradient concentration of sucrose along the sugarcane stem, with mature internodes having higher sucrose levels that decrease toward the top immature internodes. Strikingly, the bud sprouting performance along the stem follows the opposite gradient of sucrose (Whittaker and Botha, 1997; Zhu et al., 1997; Vorster and Botha, 1999; Uys et al., 2007) and the culm metabolism is apparently essential to determine the dormant status of the axillary meristem and bud outgrowth (Boussiengui-Boussiengui et al., 2016). Although few studies aimed to investigate the mechanisms responsible for successful germination/sprouting in this species (Verma et al., 2013; Singh et al., 2016; Boussiengui-Boussiengui et al., 2016), very little is known about the biochemical and molecular aspects related to this process, especially concerning sink and source interactions of the bud and culm. Plant growth and development is modulated by the balance between source and sink strengths (Paul and Foyer, 2001; Dingkuhn et al., 2007; Smith and Stitt, 2007; Patrick and Colyvas, 2014), namely production of photoassimilates in leaves and their use in non-photosynthetic organs. In sugarcane, sucrose can be quickly metabolized in sink tissues to maintain its levels within a proper range, enabling fast responses to alterations in sucrose supply and demand. However, depending on the carbon demand, the culm starts to act as an additional source tissue, mobilizing sucrose to sustain developmental transitions. In the case of sprouting, sucrose will be used to promote axillary bud outgrowth and seedling establishment (O’Neill et al., 2012). The amino acids leucine and isoleucine seem also to play a later role during this process (O’Neill et al., 2012) and isotopic analysis demonstrated that nitrogen reserves from the culms are important for seedling establishment in the first 50–60 days of development (Carneiro et al., 1995). In this sense, remobilization of carbon and nitrogen mediated by sucrose and amino acids from the culm is crucial for the establishment of a new shoot from the axillary meristem. However, it still remains to be elucidated which key metabolites participate in breaking the axillary meristem dormancy. It is known that bud outgrowth is inhibited by the action of hormones in a phenomenon termed apical dominance, which can be suppressed by excision, developmental transitions or diseases (Botha et al., 2013; Rameau et al., 2015; Barbier et al., 2017). In this work we focused on investigating how primary compounds of central metabolism, rather than hormones, behave and interact in buds and culms during sugarcane bud outgrowth. Unraveling the interaction between hormone and central metabolite signaling will be crucial to dissect the temporal cascade controlling bud outgrowth release.

As plant growth regulation is closed modulated by the primary metabolism, our study used GC-MS-based metabolomics to unravel these relationships in culm and bud among different genotypes. Due to the fact that the modern commercial sugarcane varieties have narrow genetic basis, we first addressed if metabolic features would display any degree of variability among the selected sugarcane cultivars. Statistical tests (ANOVA) on metabolome data did not only confirm metabolic variability, but also unravel differences between the studied organs.

In order to further unravel small molecules involved in this process, metabolite–metabolite correlation analysis was performed within and between tissues. Several studies pinpointed the high connectivity of amino acids in Arabidopsis, tomato and maize (Schauer et al., 2006; Toubiana et al., 2015; Wen et al., 2015), suggesting that their network is controlled by a high degree of metabolic regulation (Galili and Höfgen, 2002). Accordingly, our results showed that overall amino acids were highly correlated. Interestingly, glutamate and serine were among the few metabolites presenting correlations between culm and bud. Glutamate, a hub in amino acid metabolism, is substrate of glutamine synthetase (GS) to generate glutamine or is formed by the conversion of glutamine and 2-oxoglutarate in the presence of either reduced ferredoxin (Fd) or NADH by glutamate synthase (GOGAT) during inorganic ammonium assimilation (Lea and Miflin, 1974; Yamaya and Oaks, 2004). In rice, transgenic plants lacking the cytosolic glutamine synthetase 1;2 (GS1;2) exhibited a severe suppression of bud outgrowth (Ohashi et al., 2015), suggesting a role of glutamate as signal molecule for sensing nitrogen status and controlling this process. Furthermore, glutamate is also a precursor of serine biosynthesis in a non-photorespiratory route called phosphorylated pathway, which was the other metabolite linking culm to bud metabolism and has been shown to control cell proliferation (Cascales-Minana et al., 2013; Ros et al., 2014). In this context, metabolomics is a powerful tool for identifying candidate metabolic pathways involved in diverse biological processes.

With respect to the tissue-specific metabolic networks, the culm presented a more coordinately regulated metabolism than the bud. Due to its high concentration in parenchyma cells of stem internodes, sucrose was one of the main hubs in the culm network, as expected. This disaccharide was responsible for the most negative correlation in the network with the putrescine, an important precursor for polyamine biosynthesis. Polyamines are aliphatic nitrogen compounds that have been proposed to be involved in many processes during plant growth and development in response to environmental cues (Kusano et al., 2007; Gill and Tuteja, 2010) and are crucial for plant survival as blockage of their biosynthesis leads to lethal phenotypes (Urano et al., 2005; Ge et al., 2006). Interestingly, deletion in one of the genes encoding for the enzyme S-adenosylmethionine decarboxylases, involved in both spermidine and spermine biosynthesis, leads to a bushy and dwarf phenotype in Arabidopsis by affecting cytokinin homeostasis (Cui et al., 2010). This mutant, namely *bud2-1*, has 25% higher levels of putrescine in comparison to the wild-type. Apparently, *bud2-1* has also enhanced root growth, supporting previous work that suggests putrescine as a growth promoter (Cui et al., 2010). Cytokinin levels are controlled by auxin (IAA) during bud outgrowth via apical dominance maintenance (Müller and Leyser, 2011). In this sense, opposite to putrescine, myo-inositol displays a positive correlation with sucrose. This glycoside conjugates IAA to temporarily control its availability, being hydrolyzed to set free IAA (Kowalczyk et al., 2003). Moreover, IAA conjugates with amino acids in plants, but only few conjugates (e.g., IAA–Ala, –Leu, and –Phe) are hydrolyzed to form free IAA. IAA-Asp and -Glu are in the degradation pathway or inhibition of the IAA action as IAA-Trp (Ludwig-Müller, 2011). Myo-inositol and galacturonate pathways are interconnected for ascorbate biosynthesis (Shen et al., 2009; Zhang et al., 2009), which is necessary for cell division and elongation (Tullio et al., 1999), biosynthesis of secondary metabolites and phytohormones (Smith et al., 2007). Taken together, our results suggested that the culm metabolism encompasses a complex metabolic network and confirmed the dual function fulfilled by this tissue: its initial sink role is replaced by the novel task as a nutrient source for the emergence of a new organ or seedling during the development of axillary meristem and bud outgrowth.

As the metabolic network of the culm unravel metabolites with putative role on bud outgrowth, we next investigated whether the metabolic composition among the selected commercial cultivars could be associated with sprouting rate. Our data shows that bud metabolome solely cannot explain the differences in the sprouting rate among the genotypes. In contrast, the culm metabolome could be used to classify at least the most contrasting genotypes. Interestingly, genotypes with higher sprouting rates tended to have higher levels of certain metabolites, as it was the case of putrescine, whereas genotypes with low sprouting rates presented higher levels of sugars and amino acids, especially the branched-chain amino acids. These metabolites were positively correlated within their groups but were negatively correlated to each other in the metabolite–metabolite network. These findings suggest that carbon and nitrogen metabolism is not only involved on bud outgrowth but can also regulate this process mediating crosstalk with signaling pathways as, for example, forming conjugated compounds with phytohormones (Kowalczyk et al., 2003; Ludwig-Müller, 2011). Such approach has the potential for selecting metabolic markers and pathways associated to a certain agronomic traits in sugarcane as it was already successfully shown for other crop species (Meyer et al., 2007; Toubiana et al., 2012; Witt et al., 2012).

Our data also demonstrated that the sugarcane metabolome and bud sprouting rate are partially influenced by the genotype at least for the studied cultivars. As these commercial cultivars share part of their genetic background, we considered their genetic relatedness using pedigree information. In breeding programs, sugarcane flowering requires specific environmental conditions and it is highly genotype-dependent. Therefore, synchronization of panicles and flowering time is a challenge, and in many cases, precludes the accomplishment of desirable bi-parental crosses. In order to circumvent this limitation, multi-parental crosses can be performed as an alternative to achieve seed production. In those crosses, only the identity of the mother plant is known, and the pollens come freely from diverse male individuals. Out of the 16 genotypes used in this work, 7 presented an unknown male parental, indicating that these genotypes were obtained from multi-parental crosses. Furthermore, some genotypes, as TUC71-7, SP70-1143, RB855536 and particularly RB72454, are parental of several pedigree crosses leading to an overrepresentation of their genetic background in the selected genotypes, indicating the presence of genetic relatedness (kinship) in this panel.

Despite the incomplete record of both parentals in multi-crosses presented in this pedigree, it was partially possible to correlate bud sprouting and metabolome with the genetic information. We speculate that this correlation would be higher if more contrasting cultivars were analyzed. Even so, these results suggest that metabolic profile can be partially conserved at the parent-progeny degree in sugarcane, but not at more distant parentage levels. The use of metabolome as a proxy for genetics is appealing in sugarcane due to the complexity of its genome. Our results indicate that this approach should be feasible, opening the perspective for its application to assist sugarcane breeding programs.

## Conclusion

The work presented here clearly shows how metabolomics can be used to enrich the understanding of agronomic traits dependent on metabolic composition focusing on bud sprouting, a crucial process determining yield in sugarcane. Variability in metabolic features were identified even under a narrow genetic background typical for modern sugarcane cultivars. Metabolite–metabolite correlation analysis was performed within and between tissues in order to add information on how the metabolism of buds and culms interact to promote sprouting. Metabolic networks revealed more complex patterns for culms in relation to buds, and enabled the recognition of key metabolites (e.g., sucrose, putrescine, glutamate, serine, and myo-inositol) affecting sprouting ability. Finally, those results were associated with the genetic background of each cultivar, showing that metabolites can be potentially used as indicators for the genetic background. Analysis of association panels with broader genetic variability and the use of informative genetic molecular markers could be used in the future to confirm the predictive power of metabolomics.

## Author Contributions

DAF, MSC, and CC conceived the study. LGFA, JAA, and LDW performed the experiments. DAF, AC-G, RRA, and CC analyzed the data. MCMM, DAF, AC-G, MSC, and CC wrote the manuscript. MSC and HPH provided the supportive information.

## Conflict of Interest Statement

The authors declare that the research was conducted in the absence of any commercial or financial relationships that could be construed as a potential conflict of interest. The reviewer AF declared a shared affiliation, though no other collaboration, with one of the authors RRA to the handling Editor.

## Footnotes

1 https://www.ridesa.com.br/censo-varietal

2 http://gmd.mpimp-golm.mpg.de/

3 https://www.r-project.org/

## Supplementary Material

FIGURE S1 Pedigree of the sixteen selected sugarcane genotypes and their corresponding parentals. Gray circles represent parental genotypes that were not evaluated in this study; red, orange, blue, and green circles are genotypes ranked as low, intermediate-low, intermediate-high, and high sprouting, respectively. The arrows connecting genotypes are in the same color as their respective relatedness.

FIGURE S2 Numerator relationship matrix among selected sugarcane commercial cultivars. The colors indicate the grade of relationship from low (red) to high (blue).

FIGURE S3 Box plots of sprouting index of the sixteen selected sugarcane genotypes. For comparison among cultivars, the sprouting average considering the quartile analysis to classify them as low, intermediate-low, intermediate-high, and high sprouting was plotted. The groups are displayed in red, orange, blue, and green, respectively.

FIGURE S4 Effect of genotypes on the levels of individual metabolites. Histograms show the number of metabolites whose levels changed according to the significance indicated by *P*-values. Bonferroni-corrected ANOVA was used to evaluate the effects of genotypes on culm **(A)** and bud **(B)**.

TABLE S1 Summary of the selected sugarcane genotypes and classification of their agronomic traits. Genotypes are coded from 1 to 16 and ranked as low (red), median-low (orange), intermediate-high (blue), and high sprouting (green) ability. ^∗^POL: percentage by weight of apparent sucrose; ^1^Data obtained from RIDESA breeding program (Carneiro, personal communication). ^2^Data obtained from greenhouse experiments performed in this study.

TABLE S2 Levels of all detected metabolites in culm and bud of the selected sugarcane cultivars.

TABLE S3 Metabolite–metabolite correlations for bud, culm, and common to both tissues. Significant pairwise correlations within and between tissues (*r* ≥ 0.5, *p* ≤ 0.05) were highlighted in blue and yellow, representing positive and negative correlations, respectively.
