# Supplementary figures and images for: Metabolite Profiles of Sugarcane Culm Reveal the Relationship Among Metabolism and Axillary Bud Outgrowth in Genetically Related Sugarcane Commercial Cultivars
Source: Front Plant Sci. 2018 Jun 25;9:857. doi: 10.3389/fpls.2018.00857 (PMC6027322; doi:10.3389/fpls.2018.00857)

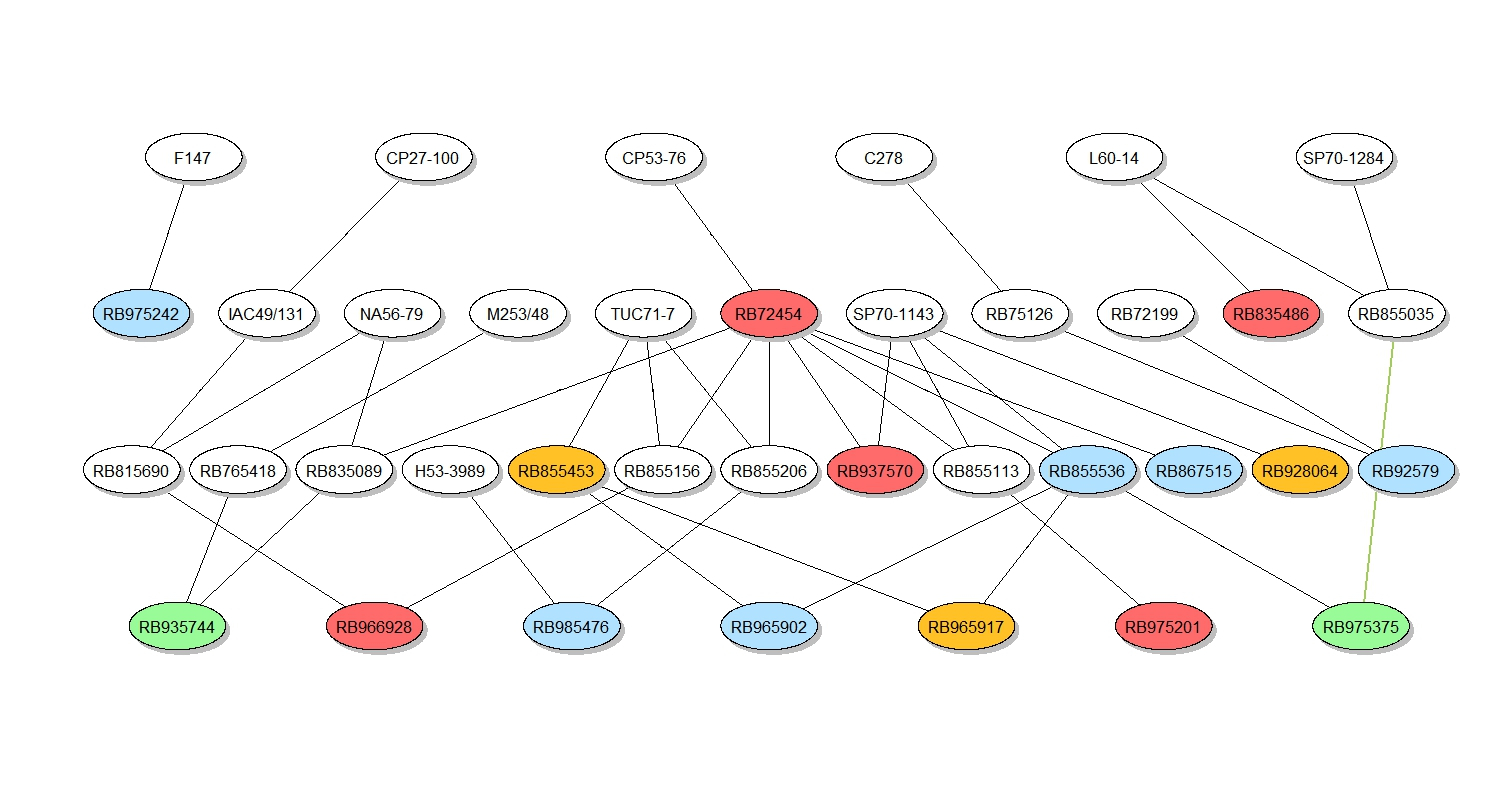

Supplement: FIGURE S1 — Pedigree of the sixteen selected sugarcane genotypes and their corresponding parentals. Gray circles represent parental genotypes that were not evaluated in this study; red, orange, blue, and green circles are genotypes ranked as low, intermediate-low, intermediate-high, and high sprouting, respectively. The arrows connecting genotypes are in the same color as their respective relatedness. [file Image_1.JPEG]

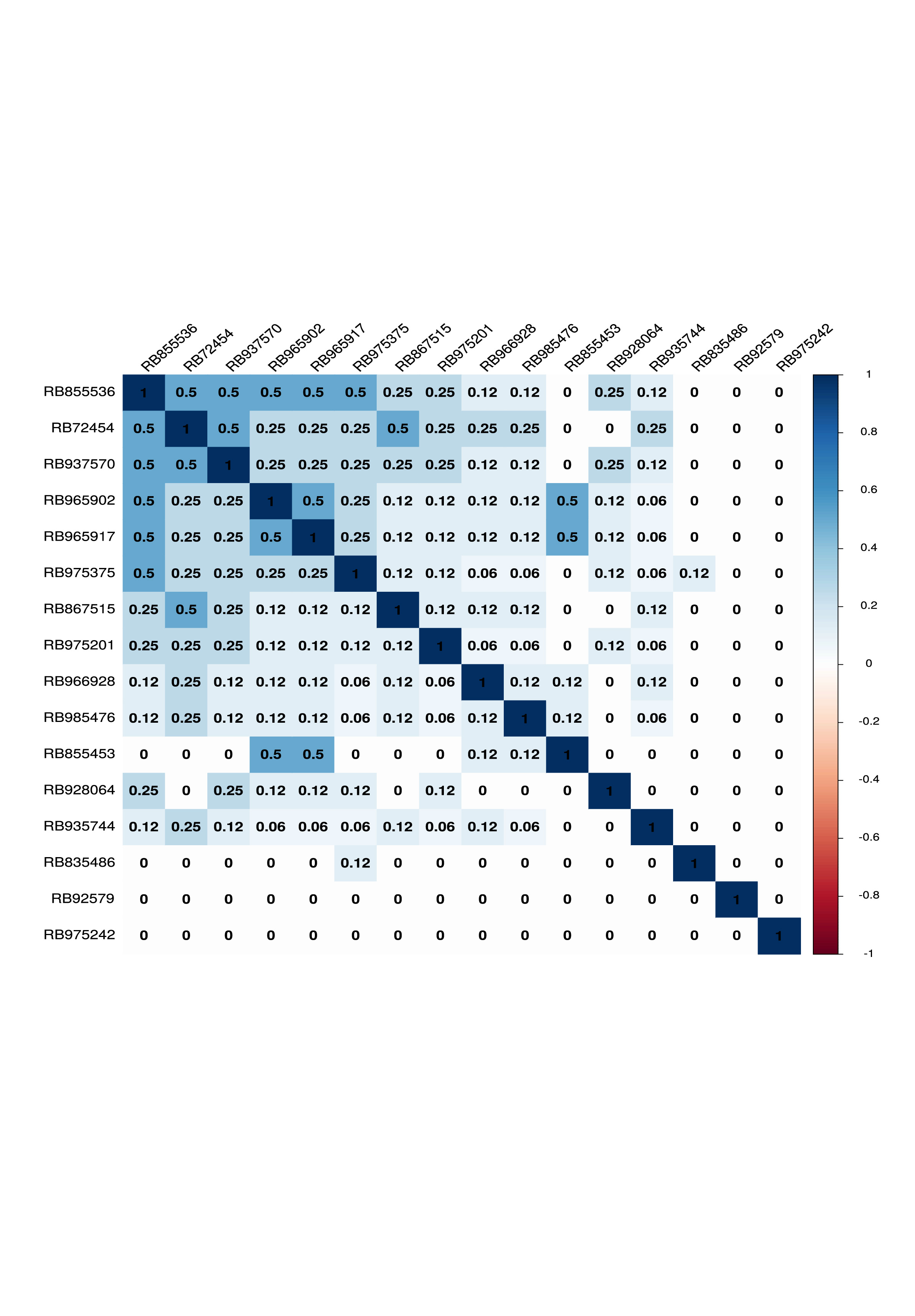

Supplement: FIGURE S2 — Numerator relationship matrix among selected sugarcane commercial cultivars. The colors indicate the grade of relationship from low (red) to high (blue). [file Image_2.JPEG]

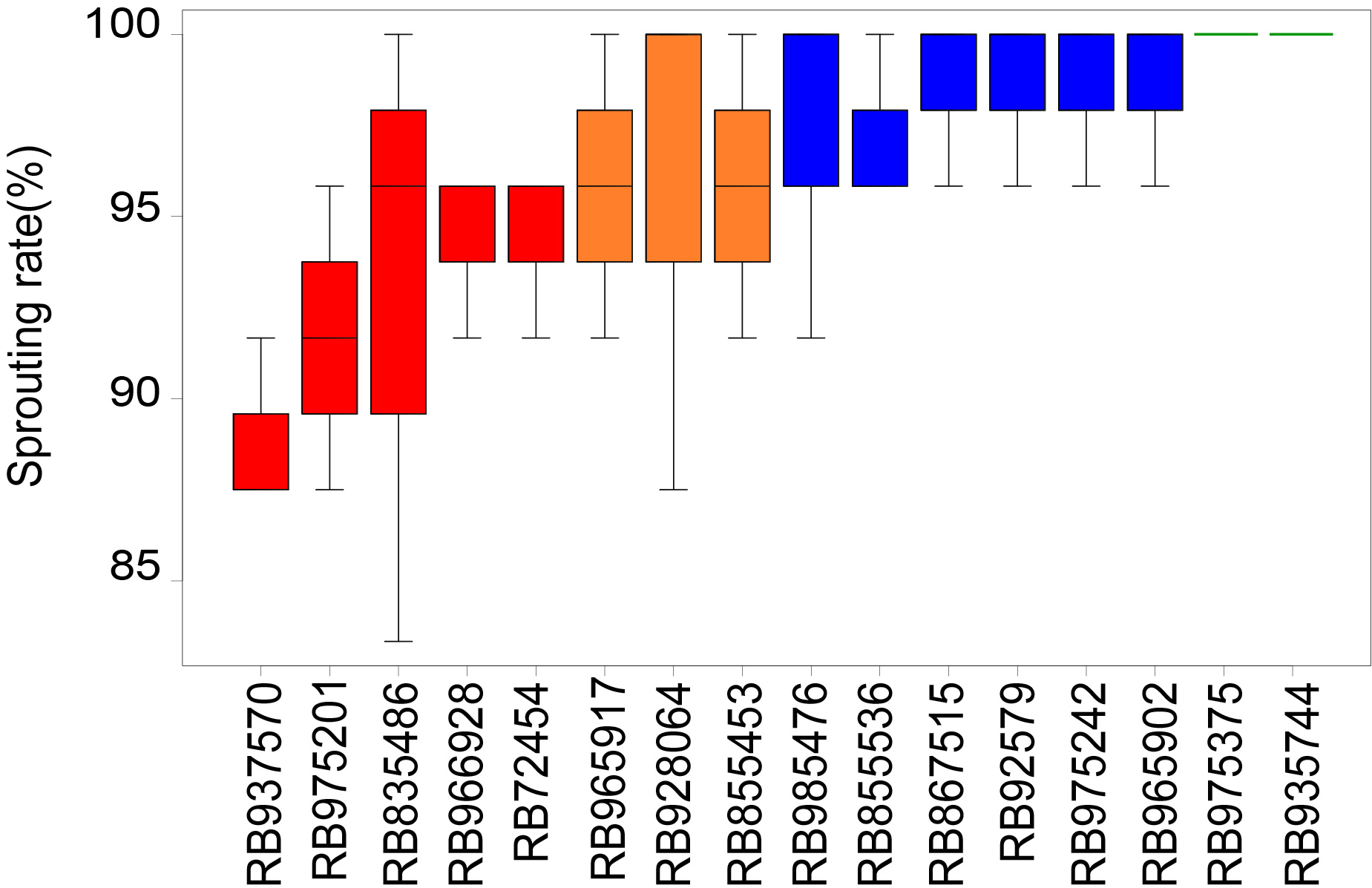

Supplement: FIGURE S3 — Box plots of sprouting index of the sixteen selected sugarcane genotypes. For comparison among cultivars, the sprouting average considering the quartile analysis to classify them as low, intermediate-low, intermediate-high, and high sprouting was plotted. The groups are displayed in red, orange, blue, and green, respectively. [file Image_3.JPEG]

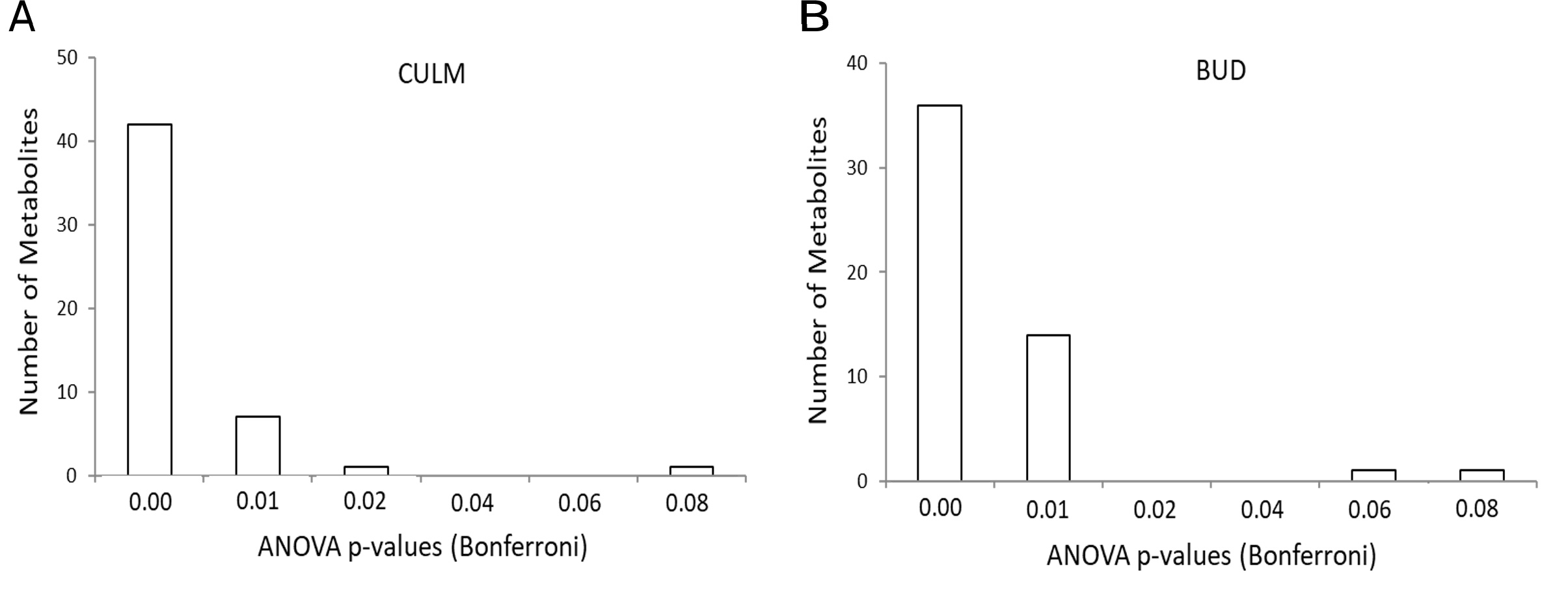

Supplement: FIGURE S4 — Effect of genotypes on the levels of individual metabolites. Histograms show the number of metabolites whose levels changed according to the significance indicated by P-values. Bonferroni-corrected ANOVA was used to evaluate the effects of genotypes on culm (A) and bud (B). [file Image_4.JPEG]
