# Supplementary material for: Metabolite Profiles of Sugarcane Culm Reveal the Relationship Among Metabolism and Axillary Bud Outgrowth in Genetically Related Sugarcane Commercial Cultivars
Source: Front Plant Sci. 2018 Jun 25;9:857. doi: 10.3389/fpls.2018.00857 (PMC6027322; doi:10.3389/fpls.2018.00857)
Supplement: TABLE S1 — Summary of the selected sugarcane genotypes and classification of their agronomic traits. Genotypes are coded from 1 to 16 and ranked as low (red), median-low (orange), intermediate-high (blue), and high sprouting (green) ability. ∗POL: percentage by weight of apparent sucrose; 1Data obtained from RIDESA breeding program (Carneiro, personal communication). 2Data obtained from greenhouse experiments performed in this study. [file Table_1.DOCX]

**Supplementary Table 1. Summary of the selected sugarcane genotypes and classification of their agronomical traits.**

| Variety | ID | Parents^1^ | Tillering (First/Second Ratoon)^1^ | Sprouting Index (Min - Max %)^2^ | POL%*^1^ | Flowering^1^ |
| --- | --- | --- | --- | --- | --- | --- |
| RB937570 | 1 | RB72454 x SP70-1143 | Medium/High | 87.5 – 91.7 | 14.5 | Frequent |
| RB975201 | 2 | RB855113 x ? | High/High | 87.5 – 91.7 | 15.9 | Rare |
| RB835486 | 3 | L60-14 x ? | Low/Medium | 83.3 - 100 | 17.2 | Eventual |
| RB966928 | 4 | RB855156 x RB815690 | High/High | 91.7 – 95.8 | 16.4 | Eventual |
| RB72454 | 5 | CP53-76 x ? | Medium/Medium | 91.7 – 95.8 | 15.4 | Eventual |
| RB965917 | 6 | RB855453 x RB855536 | High/High | 91.7 – 100 | 15.8 | Rare |
| RB928064 | 7 | SP70-1143 x ? | Medium/High | 87.5 – 100 | 15.0 | Rare |
| RB855453 | 8 | TUC71-7 x ? | Medium/Medium | 91.7 – 100 | 16.0 | Frequent |
| RB985476 | 9 | H53-3989 x RB855206 | High/High | 91.7 – 100 | 15.5 | Eventual |
| RB855536 | 10 | SP70-1143 x RB72454 | Medium/High | 95.6 - 100 | 15.9 | Absent |
| RB867515 | 11 | RB72454 x ? | Medium/Medium | 95.6 - 100 | 15.4 | Eventual |
| RB92579 | 12 | RB75126 x RB72199 | High/High | 95.6 - 100 | 15.0 | Eventual |
| RB975242 | 13 | F147 x ? | Medium/High | 95.6 - 100 | 14.5 | Absent |
| RB965902 | 14 | RB855536 x RB855453 | High/High | 95.6 - 100 | 15.8 | Absent |
| RB975375 | 15 | RB855035 x RB855536 | High/High | 100 - 100 | 16.0 | Low |
| RB935744 | 16 | RB835089 x RB765418 | Medium/Medium | 100 – 100 | 14.5 | Rare |

Genotypes are coded from 1 to 16 and ranked as low (red), median-low (orange), intermediate-high (blue) and high sprouting (green) ability.

* POL: percentage by weight of apparent sucrose;

^1^ Data obtained from RIDESA breeding program (Carneiro, personal communication)

^2^ Data obtained from greenhouse experiments performed in this study
